# A framework for building cognitive process models

**DOI:** 10.3758/s13423-020-01747-2

**Published:** 2020-07-06

**Authors:** Jana B. Jarecki, Jolene H. Tan, Mirjam A. Jenny

**Affiliations:** 1 Center for Economic Psychology, University of Basel, Petersplatz 1, 4051 Basel, Switzerland; 2 Max Planck Institute for Human Development, Lentzeallee 94, 14195 Berlin, Germany; 3 Harding Center for Risk Literacy and Center for Adaptive Rationality, University of Potsdam, Virchowstrasse, 214482 Potsdam, Germany

**Keywords:** Cognitive process model, Cognitive model, Computational model, Definitions, Marr’s levels

## Abstract

The term *process model* is widely used, but rarely agreed upon. This paper proposes a framework for characterizing and building cognitive process models. Process models model not only inputs and outputs but also model the ongoing information transformations at a given level of abstraction. We argue that the following dimensions characterize process models: They have a scope that includes different levels of abstraction. They specify a hypothesized mental information transformation. They make predictions not only for the behavior of interest but also for processes. The models’ predictions for the processes can be derived from the input, without reverse inference from the output data. Moreover, the presumed information transformation steps are not contradicting current knowledge of human cognitive capacities. Lastly, process models require a conceptual scope specifying levels of abstraction for the information entering the mind, the proposed mental events, and the behavior of interest. This framework can be used for refining models before testing them or after testing them empirically, and it does not rely on specific modeling paradigms. It can be a guideline for developing cognitive process models. Moreover, the framework can advance currently unresolved debates about which models belong to the category of process models.

**Electronic supplementary material:**

The online version of this article (10.3758/s13423-020-01747-2) contains supplementary material, which is available to authorized users.

Cognitive processes—how the mind transforms information to arrive at behavior—have been a focal topic in psychology for a century (Wundt, 1911), and have gained momentum during the cognitive revolution. Gregg and Simon (1967) advocated for “process models” as models with precise assumptions about how mental processing of information leads to behavior. In the past years, citations of database-indexed publications using the term have increased steeply, even when controlling for a positive citation trend: Fig. 1 shows that in 2018, citations of articles mentioning process models outnumbered citations of articles mentioning “formal” or “computational” models. Process model citations increased by a factor of almost 5, with a mean annual growth rate in absolute citations of 31% compared with 20% for all citations. Simultaneously, there has been a growing interest in process measures (Schulte-Mecklenbeck, Kühberger, & Ranyard, 2011a).

**Fig. 1:**
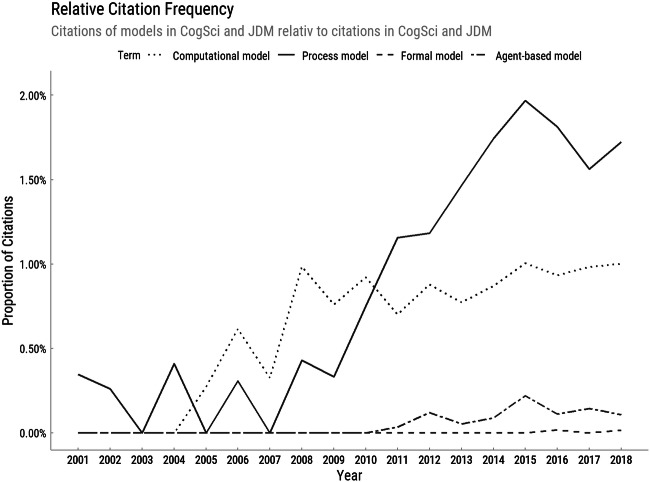
Increasing citation frequency of publications using the term “process model”. Source: Web of Knowledge, accessed May 2019. The solid line shows the proportion of citations of articles that include the terms “process model” AND “cognitive science” AND “judgment and decision making” relative to citations including the latter terms but excluding “process model.” The dotted lines depict the respective proportions for articles that include the term “agent-based model,” “formal model,” or “computational model” instead of “process model.” Cognitive science and judgment and decision making were operationalized as “cognitive,” “psychology,” AND “judgment and decision making” OR “decision making”

This trend shows that investigating mental processes is considered relevant and useful to understand human cognition by many psychologists. By “process,” we refer to the change of the state of (cognitive) systems over time (Hartmann, 1996). To date, not much advice exists on the general development of cognitive process models (see Grüne-Yanoff, 2014), besides very broad overviews (e.g., Sun, 2008), and very model-specific implementation tutorials (e.g., Griffiths & Yuille, 2008; Pothos & Busemeyer, 2013). Simultaneously, psychologists debate which cognitive models constitute process models (Brandstätter, Gigerenzer, & Hertwig, 2006). For example, do connectionist networks describe processes (McClelland et al., 2010) or functions (Griffiths, Chater, Kemp, Perfors, & Tenenbaum, 2010)? Is the recognition heuristic (Goldstein & Gigerenzer, 2002) a process model (Pohl, 2011)? Our aim in the present article is to provide a framework to clarify the requirements of cognitive process models in general. From that framework, we derive a checklist that enables researchers interested in cognitive processes to identify exactly what parts of a model need to be specified in order to arrive at predictions that are testable on a process level.

## Conceptions of process models

Experts disagree on process model properties, and the literature uses the term with different connotations.

### Disagreements between experts

We asked psychologists and cognitive modelers if 116 cognitive models constituted process models (models were selected based on a systematic review; see the Supplemental Material). Respondents were recruited through mailing lists and emails; 65 respondents completed the survey, three were excluded,^1^ leaving *N* = 62 researchers with 35 professors, 16 postdoctoral researchers, and 11 doctoral students. Most had taught methods courses (*n* = 46) and were familiar with many models; the professors, post-doctoral researchers, and students knew and classified on average 50, 49, and 40 models, respectively. Although almost all (51 of 62) agreed that process models are important, they disagreed about which models constituted process models with an inter-rater agreement of Fleiss–Cuzick’s κ = .27, far below the .60 benchmark for good agreement (Fleiss & Cuzick, 1979, p. 539). A split by seniority yielded similarly low κ values of .33, .17, and .14 for professors, researchers, and students, respectively.

This disagreement also suggests that the meta-theories related to process models like Marr’s (1982) three levels of analysis—computation, algorithm, and implementation—have not characterized the properties of process models precisely enough. Though Marr’s levels have been widely adopted (e.g., Chater, 2009; Griffiths, Lieder, & Goodman, 2015; Huang, Sen, & Szidarovszky, 2012; Jones & Love, 2011; Sanborn, Griffiths, & Navarro, 2010), their application poses difficulties (summarized in Griffiths et al., 2015). Researchers tend to locate process models at the algorithmic level, explaining “the algorithm for the transformation” (Marr, 1982, p. 25), but this fails to define process models: Asked if Marr’s algorithm level clarifies what process models are, the 38 respondents being familiar with Marr’s levels were divided between does not clarify at all (*n* = 16) and clarifies completely (*n* = 20) around a neutral midpoint (*n* = 2) on a 7-point scale.

### Disagreements in the literature

Why is there such disagreement? We think that the disagreement is because the literature lacks a clear definition of process models. Instead of referring to process models by a set of characteristics, process models are discussed with various implicit connotations.

#### Connotation 1: Process models versus rational models

Some work mentions process models in opposition to *rational models* (e.g., Bergert & Nosofsky, 2007; Chater, 2009; Jekel, Glöckner, Fiedler, & Bröder, 2012; Lee & Cummins, 2004). Such rational models provide optimal solutions to a problem (Tanner & Swets, 1954) or constrained optimal solutions (rational analysis by Anderson, 1991) to formal statistical problems faced by decision makers (Griffiths et al., 2010; Lewis, Howes, & Singh, 2014). Cognitive processes can then be singled out by the “ways in which human behavior deviates from ideal solutions” (Griffiths, Vul, & Sanborn, 2012, p. 263). From this, it is implied that process models—contrary to rational models—are models that yield solutions that are *not* optimal or only approximately optimal within a margin of error (rational process models; see Griffiths et al., 2015; Griffiths et al., 2012; Sanborn et al., 2010).

#### Connotation 2: Process models versus “as-if” models

Other work contrasts process models with as-if models^2^ (Berg & Gigerenzer, 2010; Glöckner & Betsch, 2011; Johnson, Schulte-Mecklenbeck, & Willemsen, 2008; Katsikopoulos & Lan, 2011). Berg and Gigerenzer (2010) define as-if models as models without psychological realism (for a similar argument, see Friedman, 1953). As-if models typically employ mathematical representations chosen for elegance or feasibility, are deliberately free from psychological interpretation (Brandstätter et al., 2006), and their input–output transformations need not correspond to actual cognitive processes (Glöckner & Betsch, 2011; Glöckner & Witteman, 2010). Some argue that whereas as-if models ignore cognitive capacity constraints and may include computationally complex operations, process models assume simple(r) capacity-constrained operations (V. M. Chase, Hertwig, & Gigerenzer, 1998; Gigerenzer, Todd, & the ABC Research Group, 1999). The implication is that process models need to be simple and respect capacity limits or link the proposed operations directly to psychological constructs (e.g., Myung, Pitt, & Kim, 2003).

#### Connotation 3: Formal features as a common denominator of process models

Discussions of process models have also invoked formal aspects. Early process models were linked to symbolic languages (Einhorn, Kleinmuntz, & Kleinmuntz, 1979; Gregg & Simon 1967; Newell, 1963; Simon & Kotovsky, 1963); others based on elementary information-processing principles (e.g., Bettman, Johnson, & Payne, 1990; W. G. Chase, 1978). Recently, many models have included mathematical tools that capture temporal unfolding such as random walks in sequential sampling models (Brown & Heathcote, 2008; Busemeyer & Townsend, 1993; Pike, 1973; Ratcliff, 1978), which are often called process models (e.g., Busemeyer & Johnson, 2008; McMillen & Holmes, 2006). From this, it might be implied that process models require specific formal frameworks.

#### Interim Summary

The term *process model* is widely used, but rarely agreed upon. Our brief literature review found different connotations of process models, ranging from suboptimality, and cognitive feasibility, to formal properties.

## The framework: Characterizing process models

**Fig. 2:**
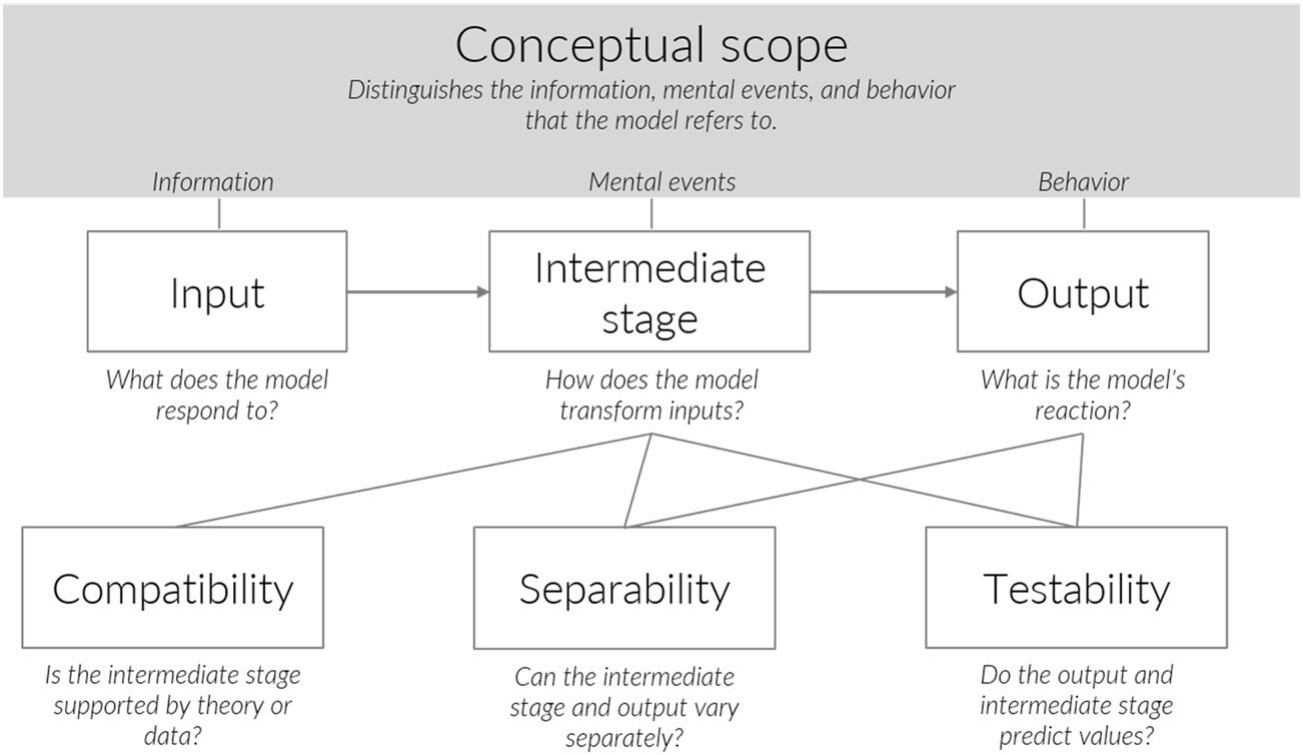
The framework for cognitive process models. The schema shows the requirements for process models: conceptual scope defining a hierarchy between the intermediate stage and the input–output level (see text), intermediate stage, compatibility, separability, and testability. Input and output are necessary for both input–output and process models. The solid lines denote the interrelatedness of the components. For details, see the text

The cognitive process model framework is a conceptual framework meant for descriptive models of cognitive processes; it is applicable to models before and after model testing. Figure 2 illustrates the process model framework and the five interrelations of the characteristics, which we will explain below. The framework proposes that process models need a clear conceptual scope, intermediate stages, compatibility, testability, and separability.

By cognitive model, we mean a graphical, mathematical, computer-programmed, or verbal stylized representation of part of the real world (e.g., Achinstein, 1965), which concerns cognitive systems in interaction with their external and internal environments. A model states assumptions about these cognitive systems, to which it is an analogy. It describes cognitive systems by the attribution of their inner structures or mechanisms from which properties of the systems can be derived. It has a particular purpose or scope (a level of abstraction; Floridi, 2008), for which it approximates cognitive systems and ignores purpose-irrelevant details (following the theoretical model in Achinstein, 1965). Models may be theory driven or used in theory development (Hartmann, 1995; Zimmermann, 1980). In general, models have an information input that is defined as observable, an information processor that transforms information, and an observable output. Process models state assumptions about processes in cognitive systems within the information processor by coherent statements, and some of these statements are defined as observable and interpreted in direct relation to cognitive systems (Carnap, 1956; for an overview of the philosophical debate, see Lutz, 2017).

### The process model characteristics

#### Process models’ conceptual scope: Nested levels of abstraction

The conceptual scope of a model describes the purpose of the model and guides interpreting model variables (Hodges, 2013). The scope defines which model variables represent which properties of the cognitive system and sets the level of abstraction. Process models have nested levels of abstraction. A level of abstraction consists of some model variables, together with the properties they represent (Floridi & Sanders, 2004); where variables are model statements, not limited to mathematical placeholders. Nested levels of abstraction imply a hierarchy among the levels in a model (see Floridi & Sanders, 2004, for a formal treatment of nested levels; Simon, 2012) such that some levels are defined to materially, temporally, or conceptually contain other levels. Concerning the framework in Fig. 2, we will refer to the variables at the more concrete nested level as intermediate level variables and the higher-level variables as input-level variables and output level variables.

To give an example for nesting, in a model of consumer choice, Gluth, Rieskamp, and Büchel (2012) define an evidence variable *e* as model input (representing values of goods), and a choice probability *P(choice | t)* as model output (representing purchase behavior at time *t*). Further, *P(choice | t)* depends on a decision variable *DV = LE(buy*_*t*_*),* which is a function of the evidence *e.* The decision variable *DV* is defined to represent a neural signal, situating *DV* is at a more concrete level than the perceived values *e* and behavioral purchases *P(choice* | *t)*. A complete specification of a cognitive model’s scope—entirely foreseeing future interpretations and applications—seems infeasible, but we advocate for clarifying a process model’s initial intended levels of abstraction and nesting relations.

#### Intermediate stages

The intermediate stage concerns the model structure and the relations among the input–output and intermediate levels, representing assumptions about how the cognitive system transforms information (Marr, 1982; Svenson, 1979; Weber & Johnson, 2009). Intermediate stages are variables at the nested intermediate level that directly or indirectly depend on the input variables and are not equal to the input variables. Intermediate stages are produced by information transformations from the input or other intermediate variables. In the real cognitive system, the process causes the phenomenon of interest; and analogously, in a process model, intermediate stages produce the higher-level output variables.^3^ While the real cognitive system can be assumed to change states continuously in time, the intermediate stage values of process models have a specific time scale, depending on the level of abstraction. Intermediate stages may change continuously in time, but may also be at a coarser temporal scale. Process models have one or more intermediate stages.

Returning to the consumer choice model example by Gluth et al. (2012), the model structure specifies that the input in terms of the evidence *e* about the value of a good influences the intermediate-stage decision value *DV,* which is sampled sequentially over time until one of two thresholds is reached, which produces the choice (= output). Intermediate stages in process models are not restricted to sequential sampling. The intermediate stage here is the value of *DV* at the points in time. Cumulative prospect theory (Kahneman & Tversky, 1979; Tversky & Kahneman, 1992) exemplifies a model without intermediate stage (cf. Pachur, Suter, & Hertwig, 2017); prospect theory computes values of risky gambles by multiplying subjective payoffs with subjective probabilities. Although the model formalizes the input–output transformation, it leaves open how transformation happens over time (e.g., if the mind first transforms payoffs, probabilities, or both simultaneously^4^). No intermediate stage is identifiable, without the refinement of prospect theory.

#### Compatibility: Differentiating process models from as-if models

Compatibility implies that the information transformations proposed in the intermediate stage of a process model are connected to current understanding of cognitive capacities. It involves detailing the cognitive assumptions in a model. Connecting model computations with cognitive functioning distinguishes cognitive models from as-if models. The purpose of compatibility is to explicate cognitive plausibility (see also Gigerenzer, Hoffrage, & Goldstein, 2008; Winkel, Keuken, van Maanen, Wagenmakers, & Forstmann, 2014), rather than placing hard restrictions on a process model’s content. Also, compatibility permits models that include new processes, if only the scientific reasoning behind the proposed processes is made explicit.

The compatibility criterion is deliberately soft. We believe, however, that it will foster model development and, importantly, theory integration. For example, imagine researchers specify a model assuming unbound cognitive capacities. Failing to relate their assumptions to the relevant literature makes the test of the processing assumptions hard. Moreover, since different models often predict the same output (model mimicry), one way to distinguish models in cases where hard empirical tests are complicated is by comparing their degrees of compatibility, given the theories behind the cognitive processes can be tested independently of the model.

Ideally, the hypothesized process is congruous with supported theories or with data about the capabilities of the modeled system. This can be a theoretical argument, an empirical argument, or a reference to data. For example, that the computations at the intermediate stage are cognitively tractable, the memory requirements do not exceed known limitations, the proposed process is consistent with empirical phenomena. To illustrate, Busemeyer and Townsend (1993) link the computations in decision field theory to findings from approach–avoidance research and choice response-time theories. Brandstätter et al.’s (2006) priority heuristic model assumes that individuals prefer one of two gambles if its payoff exceeds that of the other gamble by at least 10%, and they justify the threshold of 10% by reference to the culturally embedded decimal number system. These arguments for compatibility of the processes are verifiable independently of the performance of the process model (for a similar argument, see de Houwer, 2011).

#### Testability: Differentiating process models from machine learning tools

Testability concerns model predictions. Cognitive process models need to make testable predictions not only at the level of the output but also at the lower level of the intermediate stages (i.e., the nested level; see Scope section). Process models jointly predict values across levels. The predictions need to be specific enough to be empirically assessed using appropriate data, similar to ways in which predictions by nonprocess models, input–output models, need to be testable. Predictions at the nested level are often referred to as process predictions. Examples include predictions about attention, uncertainty, speed, and others, but what counts as process prediction is the prediction at the intermediate stage level, which is the more concrete level of abstraction. The data used to test the intermediate-stage-level predictions are often called process data (e.g., Johnson et al., 2008).

The process data is the data that the scientific community agrees on as measures of the properties of the cognitive system that the intermediate stage’s variables represent. Notably, we cannot define the class of process data in general, because the intermediate stage level that the data measures is unique to a model. However, the scope of the model and the available measurement methods together define a class of process data that is model specific. In the cognitive system, the process is the change of states over time. Because sometimes concurrent measurement is not possible, process data is often measured not while the cognitive system processes. but process data can also be assessed retrospectively, such as by confidence ratings (e.g., Schulte-Mecklenbeck, Kühberger, & Ranyard, 2011b).^5^

Besides making joint predictions for phenomena at different levels, the model predictions need to be sufficiently precise to be operationalized, tested, and measured by other researchers. These joint, precise predictions distinguish process models from machine learning tools, which need no process predictions. For process models, it suffices if, in principle, process predictions can be derived from a process model, they need not be tested yet.

One example of a model that makes a joint prediction at nested levels is the priority heuristic (Brandstätter et al., 2006), which is a computer-programmed model, that predicts choices between two risky gambles, and predicts in which order which information will be considered. The model is a decision algorithm with if–then statements based on input attributes, and is programmed such that for some attribute value combinations the algorithm exits after fewer if–then statements than for other inputs (similar to a tree depth). The exit structure is more concrete than the algorithm itself, and is thus at the lower conceptual level. The number of if–then statements depends on the input values, and makes precise, testable, ordinal response-time predictions: choices should be faster for earlier exits. The response-time prediction in the example has been tested in studies on information search, failing to support the process predictions despite support for the output predictions (Glöckner & Betsch, 2008; Johnson et al., 2008). Another example comes from the domain of forgiveness, where both Franklin’s rule and fast-and-frugal trees predicted the output (choices) well, but the (nested) information acquisition process poorly (Tan, Luan, Gonzalez, & Jablonskis, 2018). As these examples show, careful experimental design that aims to discriminate models based on their output-level predictions optimally (e.g., Myung & Pitt, 2009; Westfall, Kenny, & Judd, 2014) may not suffice to discriminate models. Comparisons of process models based on their performance on output data (e.g., choices, without considering process data) require a task that discriminates models. In case of comparing models based on output data, researchers might, however, want to refrain from drawing strong inferences about the plausibility of the underlying cognitive processes in the winning model (reverse inference), before testing the process predictions (Schulte-Mecklenbeck et al., 2011b).

#### Separability: Differentiating process models from measurement models

Separability concerns directional dependencies in the model predictions. The intermediate stage predictions produce the output predictions, but the output predictions should not fully or partially produce the intermediate stage predictions. This means that the intermediate stage variables do not decrease in their dependency on the input given the values of the output variables. In other words, knowing the output prediction does not contribute to the predictions at the intermediate stage level (reverse inference).

For instance, in Nosofsky’s (1986) generalized context model, the classification probability of stimulus *i, P(class | s*_*i*_*),* is a function of the psychological distance *d*_*ij*_ between *i* and previously experienced stimuli *j* and their class labels. The psychological distance *d*_*ij*_ is an intermediate stage. Separability means that knowledge of the classification probability *P(class | s*_*i*_*)* is not required for the computation of the distance *d*_*ij*_, but knowing the distance *d*_*ij*_ is required to compute the value of *P(class | s*_*i*_*)*.

Many models contain free parameters. If the intermediate stages consist of free parameters and no other stages, we speak of a measurement model. In measurement models, contrary to process models, the intermediate stages are a function of the output. This is exemplified by linear weighting models of multiattribute choice. These models contain attribute importance as free parameters, and the importance weights usually do not only depend on the input but are estimated from the output values and the data. The separability criterion can help to transform these models into process models by suggesting to make the importance dependent on, for example, a visual saliency model, nested in the linear-weighting model, that computes bottom-up importance weights.

The benefit of separability in process models is that the input–output relation and the input–intermediate–stage relations can be independently empirically supported. Separability means that empirical evidence can support the process predictions, while not supporting the output predictions (and vice versa). Separability can take different forms. Models may involve parameters that depend on the input and lead to process predictions. Drift rates in sequential sampling models, which yield reaction time predictions, may depend on features of the stimuli (Bhatia, 2014). In this instance, separability holds: reaction-time data and choice data can support the model independently, and the parameters here are not mere measures of reaction times. Other models lack a functional dependency between parameters and input, but the structure of the model produces separable process predictions. Classification or decision trees, for instance, can involve early exits in one branch (e.g., fast-and-frugal trees; Jenny, Pachur, Lloyd Williams, Becker, & Margraf, 2013; Martignon, Vitouch, Takezawa, & Forster, 2003). In fast-and-frugal trees, which have an exit at each level, the tree’s exit structure predicts shorter reaction times whenever decision makers reach an early exit. Critically, these trees are parameter free because the question order and the exit structure are fixed.^6^ Another class of models involves process predictions as a direct function of the model input. Consider choice models with attention weights, where the weights are a function of stimulus saliency, and yield process predictions for eye gaze. In this case, separability also holds: Process data and choice data can independently support the models’ predictions. Separability makes no prescriptions about including free parameters or not; rather, it refers to the implementation of the process predictions in a model.

The criterion of separability aims at protecting researchers from concluding that processes in models are likely from a good model performance regarding output data (e.g., choices), “affirming the consequent” (Geis & Zwicky, 2011). Social preference models in economics, for example, have been criticized by Burton-Chellew and West (2013) for inferring “the existence of prosocial preferences . . . post hoc from the results of economic games, rather than with direct experimental tests” (p. 216). While affirming the consequent is unproblematic for output models (Friedman, 1953) or measurement models, in process models it renders the inferences we draw about the actual process implausible.

Separability can also be useful to refine models. For example, random walk-based models (e.g., Ratcliff & Rouder, 1998) predict choices and reaction times, given the model parameter. Early versions of random walk models predicted choices relatively well, but did not always capture response times. Some versions predicted equal reaction times for correct and incorrect choices in inference tasks (Ratcliff & Tuerlinckx, 2002), while in the data response times tend to be faster for errors than for correct responses (Ratcliff & Smith, 2006; Ratcliff, Van Zandt, & McKoon, 1999). As a result, the parameters in the random walk model could be refined. The random walk models used separability to simultaneously allow response-time predictions to be tested against response-time data, and choice predictions against choice data. Without separability, this discrepancy could have gone unnoticed (for a similar argument, see also Gregg & Simon, 1967). Although not every model including a random walk fulfills separability, the example illustrates how separability is useful in model refinement.

### Summary and comments on the framework

In sum, a process model should have a clear scope and contain at least one cognitively motivated intermediate stage that occurs after the input but before the output. The model should also yield separate predictions for the processes and for the behavior at the output level, allowing the two stages to be empirically disentangled. The process indicated by the intermediate stage should be compatible with mental capacities and be empirically testable.

Our framework does not imply that process models are better models than output models. A model that fulfills the process model criteria can obviously be falsified (for a discussion of what constitutes a good model, see e.g., Myung et al., 2003), and we wish to stress that model performance and the nature of process models are two separate discussions.

The process model framework includes testability, separability, and compatibility—and no other characteristics—for the following reasons. The characteristics are deliberately independent of formal model notation (stochastic vs. deterministic, verbal vs. statistical, parallel vs. serial, etc.) because form and content are independent. Researchers can choose the form of a process model. Throughout the article, we deliberately provided examples from different formal modeling paradigms. Second, the requirements testability, separability, and compatibility indirectly or directly link the model to data, which we consider paramount. Third, our framework circumvents the issues of optimality and suboptimality mentioned in the Introduction, which relate more to choosing between optimality criteria than to the model class. Last, the framework provides criteria that are independent of a particular theory.

The proposed process model framework can be used by any scientist aiming to build new process models. In particular, the process model criteria are useful in the following ways: Comparably to the standardized way in which the “method” section in scientific articles is structured, a standardized structure for modeling sections will facilitate scanning the growing number of modeling publications. Considering the growing number of modeling publications, this seems useful. Further, a standardized set of criteria will facilitate differentiating models. In particular, the criterion of separability provides a precise instruction for ways to refine existing nonprocess models. Next, the criterion of compatibility will make it easier for future researchers to find the relation between theories, data, and formal models. Lastly, the separability criterion sets apart process models from other type of models.

### Application of the framework

Below we provide an example illustrating the usage of the framework with a model we consider unambiguous—a lexicographic heuristic model of decisions in a mini-ultimatum game (Hertwig, Fischbacher, & Bruhin, 2012). The model (henceforth LEX) is a graphical model (decision tree) with a depth of three (three decision nodes) and an exit node at each level. The decision nodes use three aspects of the offer that the proposer selected (relative size, possible size, own preference) as follows: Node 1: If the relative size of the offer is greater than the proposer’s share, accept it (exit), else go to decision Node 2. Node 2: If the offer is the larger of the two possible offers, accept it (exit), else proceed to decision Node 3. Node 3: If the offer is the one that the responder would have made if roles were reversed, accept, else reject. Is LEX a process model according to our framework?

#### Conceptual scope

A conceptual scope is given if the authors define not only input and output but also the properties of the cognitive system that the model variables relate to and hierarchical levels of abstraction. In LEX, the input is the proposer’s offer, the output is the acceptance or rejection decision, and the intermediate stage is related to a “social motive” like inequality aversion or kindness (Fischbacher, Hertwig, & Bruhin, 2013). The structure of the decision tree provides a more detailed temporal representation of the information transformation of the attributes of the offer compared with only the offer and the choices. Thus, LEX has a conceptual scope.

#### Intermediate stage

LEX postulates that responders evaluate offers by considering three attributes of the offer sequentially, the attributes produce the choice and the structure of the tree postulates that given different attribute combinations, a different number of decision nodes is needed, which is an intermediate variable value that depends on the input; and thus the model includes three intermediate fairness-driven decision-making stages.

#### Testability

Testability holds if models allow specific predictions to be derived for output and intermediate stages. The specification of LEX makes for two types of predictions—namely, responders’ decisions (output prediction) and an increase in response latency in the number of attributes considered (intermediate stage prediction). The predictions are precise and lie within the scope of the model; testability is fulfilled.

#### Separability

Separability holds if models predict processes without reverse inference from the output. LEX predicts response latency independently from decisions. The model might correctly predict decisions but fail to predict reaction times, or vice versa. LEX’s process and output are separable.

#### Compatibility

LEX is compatible if the intermediate stages are explicitly linked to supported theory or data regarding the conceptual scope. Fischbacher et al. (2013) reference research on the use of use similar lexicographic choice strategies (e.g., Gigerenzer & Goldstein, 1996). Therefore, compatibility is fulfilled.

We conclude that this graphical model, the LEX model, is a process model.

#### Application of the framework to ambiguous cognitive models

We now apply the framework to two models considered ambiguous process models because half of the survey respondents (see Introduction) classified them as process models: the *anchoring and adjustment model* (Tversky & Kahneman, 1974), and the *equal weighting model* (Dawes, 1979). Table 1 shows the features and the resulting classification according to the framework. Although we conclude that the models do not qualify as process models, the process model framework shows a road map as to how to convert them into process models—for example, by detailing the intermediate stages of the anchoring and adjustment model in a more explicit way. In the Supplement we apply the framework to a formal categorization model (Lamberts, 1998).

**Table 1:** Classification of two ambiguous models using the process model framework

Dimension	Anchoring and adjustment model (Tversky & Kahneman, 1974; KT74)	Equal weighting model (Dawes, 1979; D79)
Scope	*Yes.* “people make estimates by starting from an initial value that is adjusted to yield the final answer” (KT74, p. 1128). Inputs to the models are initial values; outputs are value estimates; the intermediate level that is temporally between input and output is the adjustment.	*No.* The author defines the model variables as paramorphic representations of cognition: “Hoffman termed the use of linear models a paramorphic representation of judges, by which he meant that the judges’ psychological processes did not involve computing an implicit or explicit weighted average of input variables” (D79, p. 574, see also Dawes & Corrigan, 1974)
Intermediate stage	*Yes.* The adjustment produces the output: “estimates . . . are biased toward the initial value” (KT74, p. 1128). The intermediate stage is the adjusted value that is biased towards the anchors.	*No.* Because there is no intermediate level, there are also no intermediate stages.
Testability	*No.* The model does predict an effective outcome given a stimulus (i.e., that “different starting points yield different estimates”; KT74 p. 1128). The model includes no testable process predictions, because how much exactly the adjustment is carried out is not specified explicitly.	*No.* The model predicts an outcome (i.e., judgments), but makes no process predictions.
Separability	*No.* Since the model specifies no process predictions, data cannot support process predictions separately from outcome predictions.	*No.* Since the model specifies no process predictions, data cannot support process predictions separately from outcome predictions.
Compatibility	*Yes.* Literature is cited that shows that “adjustments are typically insufficient” (KT74, p. 1128).	*Yes.* Literature is cited that shows that “it is not in the ability to integrate information that people excel” (D79, p. 573).

*Note.* Classification was based on the information in the following publications: anchoring and adjustment model: Tversky and Kahneman (1974); equal weighting model: Dawes (1979)

## General discussion

We proposed a framework for characterizing and building cognitive process models. We argued that a process model should include at least one intermediate stage between input and output, and a conceptual scope that clarifies what the model’s input, output, and intermediate stage refer to and specifies nested levels of abstraction. A process model should provide testable hypotheses within the scope for the output and process, and, moreover, predict process data independently of its output predictions (avoiding reverse inference). Finally, the proposed intermediate stage should be compatible with current knowledge about cognition (within the scope).

Conceptual clarity about the meaning of frequently used terms is desirable in its own right, but clarity also facilitates the advancement of the area of interest. Many arguments have been made about the advantages of process models (e.g., Berg & Gigerenzer 2010; Gregg & Simon 1967) and interest in process models is growing. Yet the field provides little advice on how to build them. We think this is the result of a lack of clarity. Cognitive models that fail to meet the criteria for being considered a process model could be called “formal cognitive models.”

### Building process models

**Fig. 3:**
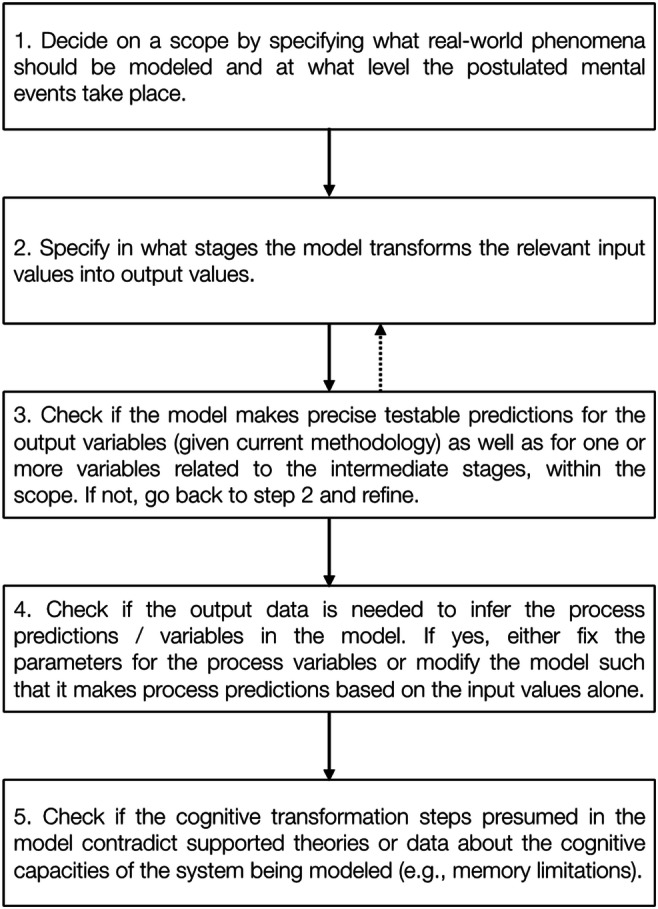
Checklist to construct cognitive process models. For further details, see text

The checklist nature of our framework enables researchers interested in cognitive processes to identify exactly what parts of a model need to be tweaked or added in order to arrive at predictions that are testable on a process level (see Fig. 3).

### Implications for process tracing

Process data are required to test a process model (Johnson et al., 2008), but it is unclear what counts as process data. For example, eye movements could be process data to decision scientists (Lemonnier, Brémond, & Baccino, 2014; Orquin & Mueller Loose, 2013), but output data to researchers studying reading (e.g., Reichle, Rayner, & Pollatsek, 2003). The separability and testability criteria of our framework can help identify process data as data that support the proposed intermediate stage. If one model is specified as a process model according to this framework, the data that the model predicts from its intermediate stages (e.g., eye movements) constitute the process data. If another researcher proposes another model with an intermediate stage that also predicts eye movements, the process predictions of these models can be compared, using process-tracing methods. Process models can connect process tracing and cognitive modeling.

### Implication for scientific debates

Our framework may advance ongoing debates about process modeling. One such debate is a normative debate, questioning the usefulness of process models, with some arguing that, given that the mind is the object of interest, models should incorporate real mental processes to provide a genuine explanation (Berg & Gigerenzer, 2010), and that process models are more realistic models of the mind than other models (Berg & Gigerenzer, 2010; Gigerenzer, 2010; Svenson, 1979). Others argue that rational models tend to describe the mind better than process or mechanistic models (e.g., Chater, 2009). The other debate concerns model classification: What counts as a process model of decision making (Ayal & Hochman, 2009; Brandstätter et al., 2006; Busemeyer, Pothos, Franco, & Trueblood, 2011; Pachur, Hertwig, Gigerenzer, & Brandstätter, 2013)? To advance these debates, a first step is providing clarity about what process models are. Once the field agrees on the characteristics of process models, researchers will be able to argue whether a model serves its intended purpose and whether a model claiming to be a process model provides the explanation that it advertises using a common language.

### Implications for model testing

Because of the separability criterion of our framework, process models should be tested with at least two sources of data—for example, choice and brain data. Critically, if models fail regarding choice data but not process data, the implications differ from cases where models fail regarding process data but not choice data. Failing to predict process data while predicting choice data well means that mainly the intermediate stage of the model needs rethinking. By contrast, failing to describe the choices while describing process data well leaves open which part of the model needs improvement. Also, in a model comparison, where one model outperforms another regarding choices, but the second outperforms the first regarding process, we may think of merging the two models.

### Implications for plausibility arguments

The assertion that process models need to have “plausible” processes was meant to constrain the space of models, but it has resulted in many degrees of freedom for the researcher. For example, Bayesian cognitive models may be plausible to some but not others (e.g., Jones & Love, 2011). Our framework defines plausibility operationally: being compatible with a supported theory or set of data. This allows third-party verification of the notion of plausibility. It additionally allows formerly plausible models to become implausible with scientific advancements. The plausibility of process models can, and we believe should, be able to change with scientific progress.

## Conclusion

The increasing use of modeling techniques is one of the most exciting trends in cognitive science. Modeling allows cognitive processes to be specified and tested at a resolution far greater than before. In particular, process modeling can foster greater understanding by testing theories and integrating diverse perspectives in order to build a full picture of human cognitive functioning. If the field is to take advantage of the explanatory potential of process models, there needs to be clarity about what constitutes a process model. We hope that our framework contributes by providing a common ground for discussions between researchers who share interest in process explanations but have backgrounds in different paradigms, so that better process models will result.

## Electronic supplementary material

ESM 1 (DOCX 4352 kb)

## Footnotes

1 One for insincere responses, two for a lack of expertise.

2 As-if models are also called paramorphic, phenomenological, or input–output models (Hoffman, 1960; Luce, 1995).

3 The intermediate stages may be the only variables that the ouput depends on, or the output may depend additionally on more input variables.

4 Although the original prospect theory formulation mentioned an editing stage before an integration stage, this temporal order was not included in the final model.

5 What types of data count as process data is model-specific and dynamic, because it depends on what psychometrics and physics regard as valid and reliable measure of the changing states of the cognitive system that the scope defines as related to the intermediate variables in the model. What counts as process data is defined by the scope of a model (see Scope section).

6 Also, in the case of continuous variables, the thresholds at which to decide “yes” or “no” in a fast-and-frugal decision tree can be fixed.
