# Supplementary material for: A framework for building cognitive process models
Source: Psychon Bull Rev. 2020 Jul 6;27(6):1218–29. doi: 10.3758/s13423-020-01747-2 (PMC7704479; doi:10.3758/s13423-020-01747-2)
Supplement: Supplementary file 1 — (DOCX 4352 kb) [file 13423_2020_1747_MOESM1_ESM.docx]

# Supplement A

# Description of the Systematic Literature Search for Models

The list of 116 models used in our survey emerged from a systematic literature search.

The search proceeded in two steps (1) we identified relevant articles that test or develop models, and (2) we extracted the models tested in these articles, and selected the most prominent judgment and decision making models.

**Step 1: Identification of Articles.** Specifically, we searched for important or new articles proposing a model (i.e., articles that had been cited more than 100 times or were published in 2004 or later, respectively) in two databases: Google Scholar and ISI Web of Science. We combined the fixed search term “model of” with synonyms for “decision making.” The precise search phrase reads *“model of * decision” | “model of * decisions” | model of * choice” | “model of * choices” | “model of * preference” | “model of * preferences” | “model of * inference” | “model of * inferences”* where *|* denotes the Boolean OR and *** can be any word.

To ensure source relevance, we restricted our search to *Judgment and Decision Making Journal*, *Psychological Review*, *Journal of Experimental Psychology (JEP): General*; *JEP Learning, Memory, and Cognition*, and *JEP Human Perception and Performance*. This yielded 433 results. From these, we first selected all articles testing cognitive models and excluded, for example, articles proposing measurement scales or mentioning but not testing models. Of these, the first author (JBJ) selected articles on judgment and decision making, excluding, for example, articles purely about perception. As this step was somewhat open to interpretation, we randomly selected a subset of 50 articles and checked the reliability of the categorization to fields by cross-coding them (coders were JBJ, JHT); this analysis showed a high level of agreement (Cohen’s kappa = .831). We therefore kept the selection by JBJ.

This procedure identified articles from the judgment and decision-making literature which dealt with models. These articles served as the sources for the models.

**Step 2: Model Extraction from the Articles.** We extracted the names of all models tested in the articles and looked up the models’ original sources (determined by their earliest

occurrence in a peer-reviewed journal or book). Importantly, model selection was independent of labels such as “process model” or “computational model.”

Contemporary decision science uses—in total— 172 individual models to explain human decision making. To ensure that the models were still relevant, we included models that had been cited more than 388 times in total or more than an average of 6.6 times per year (388 is the 66th percentile cutoff of all citations, and 6.6 is the 33rd percentile cutoff of average citations per year). We were left with 116 models.

# Supplement B Full list of Models

List of decision science models and the references included in the expert survey.

**Model Name (alphabetic order) Source**

ACT-IF, Adaptive Control of Thought in Information

Foraging (Pirolli & Card, 1999)

Additive Difference Model (Morrison 1962; Tversky, 1969) Additive Trade-off Model between Informativeness and

Accuracy (Yaniv & Foster, 1995)

Additive-utility Model of Delay Discounting (Killeen, 2009)

(DeLosh, Busemeyer, McDaniel, 1997; Busemeyer, Byun, DeLosh,

ALM, Associative Learning Model

Anchoring and Adjustment Model

ASCM, Adaptive Strategy Choice Model (of algebraic

McDaniel, 1997)

(Kahneman & Tversky, 1982; Einhorn & Hogarth, 1985)

strategies) (Siegler & Shipley, 1995)

Associative Accumulation Model (Bhatia, 2013)

Attractor Model of Visual Discrimination (Wang, 2002)

Availability Heuristic (Tversky & Kahneman, 1973) Beta Delta Preference Model of Temporal Discounting (Laibson, 1997)

Biased Encoding Model (associative storage network

model of memory-based judgment) (e.g., Hastie 1980)

Brunswik's Lens Model (Brunswik, 1956)

(Wallsten, Pleskac, & Lejuez,

BSR, Bayesian Sequential Risk Taking Model

Causal Bayes Nets

2005; Pleskac, 2008) (Spirtes, Glymour,

& Scheines, 1993; Pearl, 2000)

Complement Model of Charitable-giving (Bernheim, 1994)

Conditional Probability Model (Oaksford, Chater, & Larkin, 2000)

Constructed-Choice Model (Krantz & Kunreuther, 2007) (Graesser, Singer, & Trabasso,

Constructionist Theory of Inference Generation

1994)

CPT, Cumulative Prospect Theory (Tversky & Kahneman, 1992)

Delta-Rule Model (Rescorla & Wagner, 1972)

Denrell's Experience Sampling Model (Denrell, 2005)

(Kornblum, Hasbroucq, & Osman,

Dimensional Overlap Model

Dimensional Weight Model

1990)

(Birnbaum & Stegner, 1979; Tversky, Sattath, & Slovic, 1988)

Discrete-Slot Model of Working Memory (Zhang & Luck, 2008, 2009) DM, Diffusion Model (Ratcliff, 1978)

(Verschueren, Schaeken, &

Dual Process Model of Deductive Inference

d'Ydewalle, 2005)

EBA, Elimination by Aspects (Tversky, 1972) EBM, Frequency-sensitive Exemplar Model (Nosofsky, 1988)

EBRW, Exemplar-based Random Walk Model (Nosofksy & Palmeri, 1997)

EGCM-RT, Extended Generalized Context Model for

Reaction Times (Lamberts, 2000)

EGCM, Extended Generalized Context Model (Lamberts, 1998) EW, Equal Weighting Model (Dawes, 1979)

Exemplar Model (Medin & Schaffer, 1978) (Nosofsky, Kruschke, McKinley,

Exemplar-Based Network Model

1992)

Exponential Strategy Selection Model (Rieskamp & Otto, 2006) Extension of the Leaky, Competing Accumulator Model (Usher & McClelland, 2004) FA Model, Fractional Adjustment Model (Weber, Shafir, & Blais, 2004) Feedforward inhibition model (Shadlen & Newsome , 2001)

Forgetting Strategy Selection Model (Rieskamp & Otto, 2006) (Hancock, Masalonis, &

FSDT, Fuzzy Signal Detection Theory

Parasuraman, 2000)

GCM, Generalized Context Model (Nosofsky, 1986)

(Medin & Schwanenflugel, 1981;

General Linear Classifier

Ashby & Gott, 1988)

GQC, General Quadratic Classifier (Ashby & Gott, 1988; Ashby 1992) gRAT, Generalized Version of a Rational Model/WADD (Nosofsky & Bergert, 2007)

gTTB, Generalized Version of Take-the-Best (Nosofsky & Bergert, 2007) Herrnstein's Matching Law (Herrnstein, 1961)

(Thomas, Dougherty, Sprenger, &

HyGene

Harbison, 2008)

Hyperbolic Discounting Model (Elster, 1979)

Imagination Strategy Selection Model (Rieskamp & Otto, 2006) (Hastie & Kumar 1979; Hastie,

Incongruity-biased Encoding Model

1980, 1984; Scrull, 1981)

Increasing Probability Model (Wallsten, Pleskac, Lejuez, 2005) (Anderson & Hubert, 1963;

Independence Model of Memory and Judgment

Anderson, 1981)

Independent Race Model (Logan & Cowan, 1984) Integrated System Model of Attention and Decision

Making (Smith & Ratcliff, 2009)

LBA, Linear Ballistic Accumulator Model (Brown & Heathcote, 2008) LCA, Leaky, Competing Accumulator Model (Usher & McClelland, 2001) Leaky Accumulator Model with Relative Criteria (Ratcliff & Smith, 2004) Leaky Accumulator Model (Ratcliff & Smith, 2004)

Lexicographic Semiorder (Luce, 1956; Tversky, 1969)

Linear Decision-boundary Model (Ashby & Townsend, 1986)

Linear Regression NA

LISA, Learning and Inference with Schemas and

Analogies (Hummel & Holyoak, 1996; 1997)

Least Mean Square Network Model/Configural Cue

Adaptive Network Model (Gluck & Bower, 1988)

Matching Heuristic (Dhami & Ayton, 2001)

(Roe, Busemeyer, & Townsend,

MDFT, Multialternative Decision Field Theory MIN, Minimalist

2001)

(Gigerenzer & Goldstein, 1966; Gigerenzer et. al, 1999)

Minimum-distance Classifier (Ashby & Townsend, 1986) (Regenwetter, Dana, & Davis-

Mixture Model of Transitive Preferences

MMN, Max-minus-next Diffusion Model

Stober, 2011)

(Ratcliff & McKoon, 1997; McMillen & Holmes, 2006)

Mutual Inhibition Model (Usher & McClelland, 2001) (Czerlinski, Gigerenzer, &

Naive Bayes Classifier

Goldstein, 1999)

Nonstationary Process Increasing Probability Model (Wallsten, Pleskac, Lejuez, 2005) OU, Ornstein-Uhlenbeck Diffusion Model (Busemeyer & Townsend, 1993) PCS, Parallel Constraint Satisfaction Model for

Probabilistic Decision Tasks (Glöckner & Betsch, 2008) (Brandstätter, Gigerenzer, &

PH, Priority Heuristic

Hertwig, 2006)

Preference for Sequences Model (Loewenstein & Prelec, 1993)

Present-value Comparison Model (Ainsly, 1992)

Pretree, Preference Tree (Tversky & Sattath, 1979)

Priority Model (Rieskamp, 2008)

Probabilistic Contrast Model (Cheng & Novick, 1990)

Prototype Model (Reed; 1972)

PT, Prospect Theory (Kahneman & Tversky, 1979) (Busemeyer, Pothos, Franco, &

Quantum Judgment Model

Trueblood, 2011)

r-Model (Hilbig, Erdfelder, & Pohl, 2010)

(Birnbaum & Stegner, 1979;

RAM, Rank Affected Multiplicative Model

Random Walk Model

Birnbaum & McIntosh, 1996) (Stone, 1960; Laming, 1968; Link &

Heath, 1975)

Recruitment Model (LaBerge, 1992)

RELAC, Reinforcment Learning of Cognitive Strategies (Erev & Barron, 2005)

(Goldstein & Gigerenzer, 1999,

RH, Recognition Heuristic

2002)

Rule Competition Model (Busemeyer, & Myung, 1992)

Rule-based Categorization Models (Nosofsky, Clark, & Shin, 1989) (Nosofsky, Palmeri, & McKinley,

RULEX, Rule-plus-exception Model

SAMBA, Selective Attention, Mapping, and Ballistic Accumulation Model

SDT, Signal Detection Theory

SEMAUT, Subjective Expected Multi-Attribute Utility

1994)

(Brown, Marley, Donkin, & Heathcote, 2008)

(Tanner & Swets, 1954; Swets, Tanner, & Birdsall, 1961)

Model (Savage, 1954)

(Lu, Yuille, Liljeholm, Cheng, &

SS Power Model

Holyoak, 2008)

SSL, Strategy Selection Learning Theory (Rieskamp & Otto, 2006)

Stationary Process Model (Wallsten, Pleskac, Lejuez, 2005)

Story Model (of juror decision making) (Pennington & Hastie, 1986, 1988)

Structural Equation Model NA

(von Neumann & Morgenstern,

Subjective Expected Utility Model

1947)

SUSTAIN, Supervised and Unsupervised Stratified Adaptive Incremental Network

(Love & Medin, 1998; Love, Medin

& Gureckis, 2004)

SVM, Sequential Value Matching (Johnson & Busemeyer, 2005)

Target Model (Wallsten, Pleskac, & Lejuez, 2005) TAX , Transfer of Attention Exchange Model (Birnbaum & Stegner, 1979)

Three-stage model (Hasbroucq & Guiard, 1991)

Tradeoff Model of Intertemporal Choice (Scholten & Read, 2010)

TTB, Take-the-best (Gigerenzer & Goldstein, 1996) UCIP, Unlimited Capacity Independent Parallel

Processing Model (Townsend & Wenger, 2004)

Utility Functions 0

(Payne, Bettmann, & Johnson, 1993;

WADD, Weighted Additive Model

Keeney & Raiffa, 1976)

Warm Glow Model of Charitable-giving (Andreoni, 1990)

Weighting Model NA

(Stone, 1960; Laming, 1968; Link &

Wiener Diffusion Model

Heath, 1975)

Wyer and Srull's Storage Bin Model (Wyer & Srull, 1986)

# Supplement C Survey of Scientists with Modeling Experience Section 1.


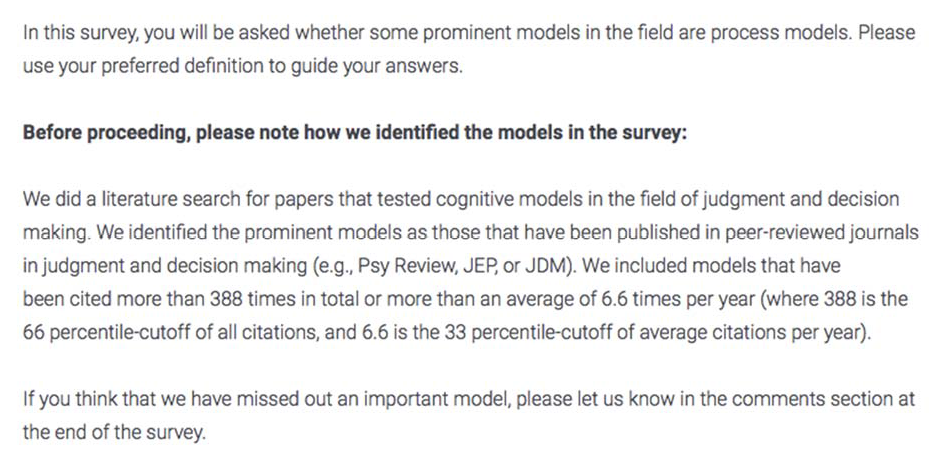


# Instructions

**Section 2. Categorization of Models**

*Note. Survey respondents responded to a total of XX models.*


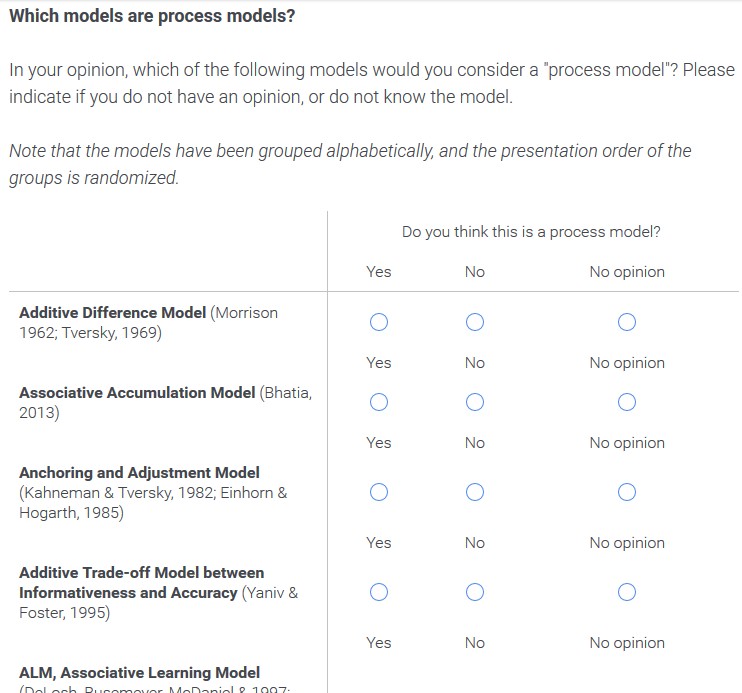

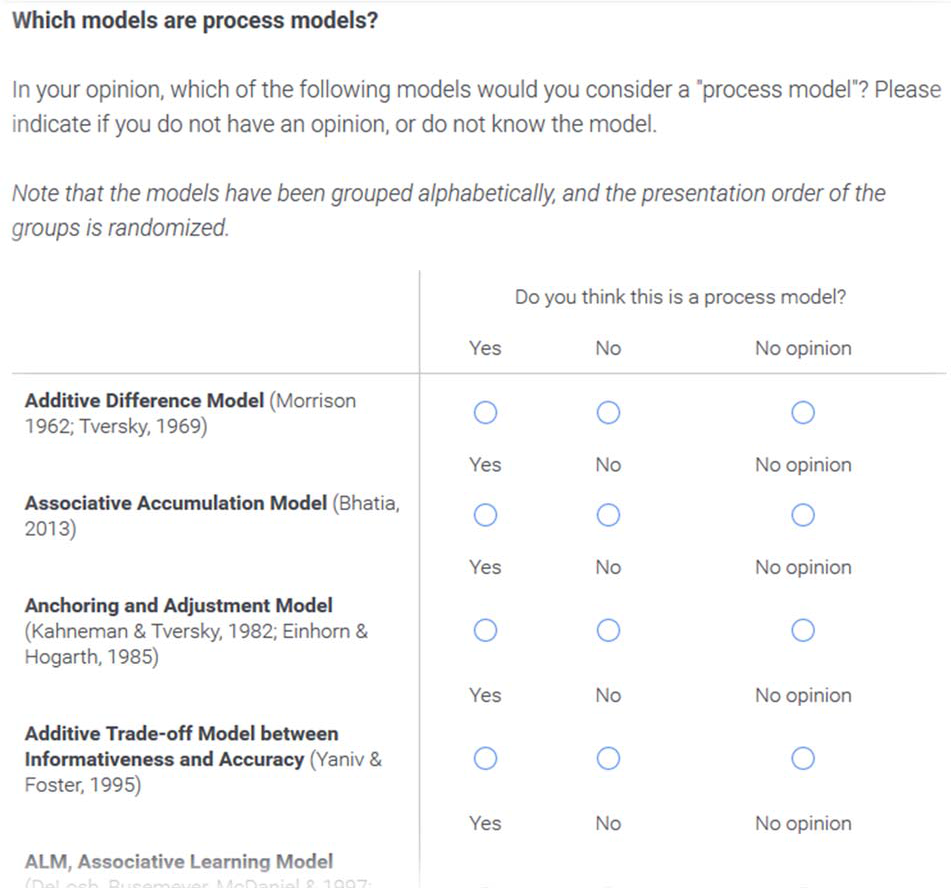


## Section 3. Familiarity with Marr’s (1982) Three Levels


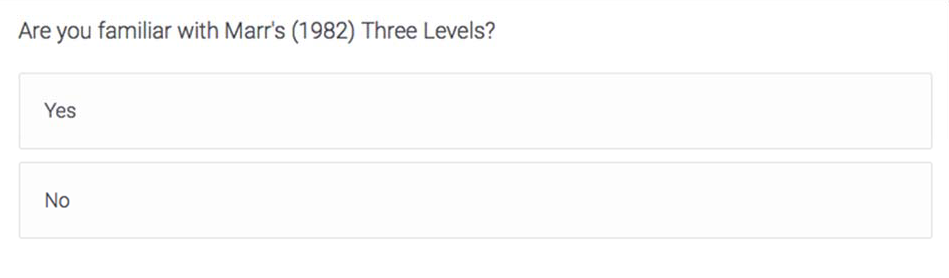


*Only if Yes:*


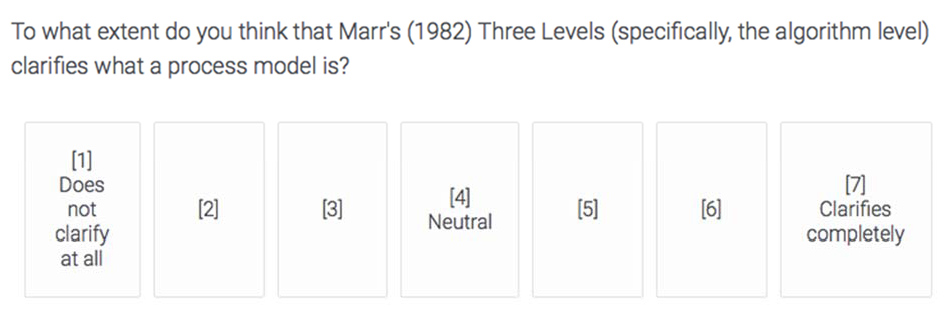


## Section 4. Opinion about Process Models


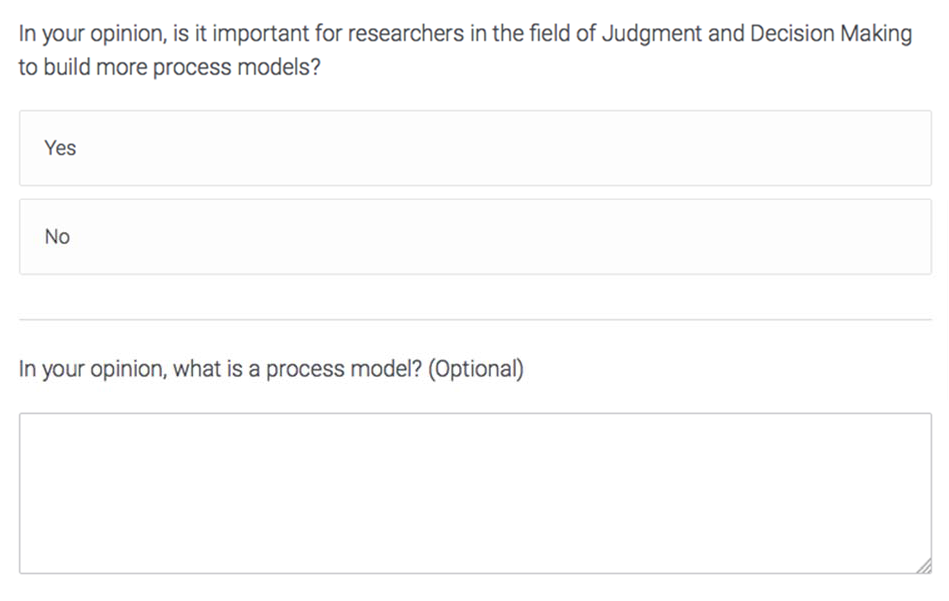


## Section 5. Background and Discipline


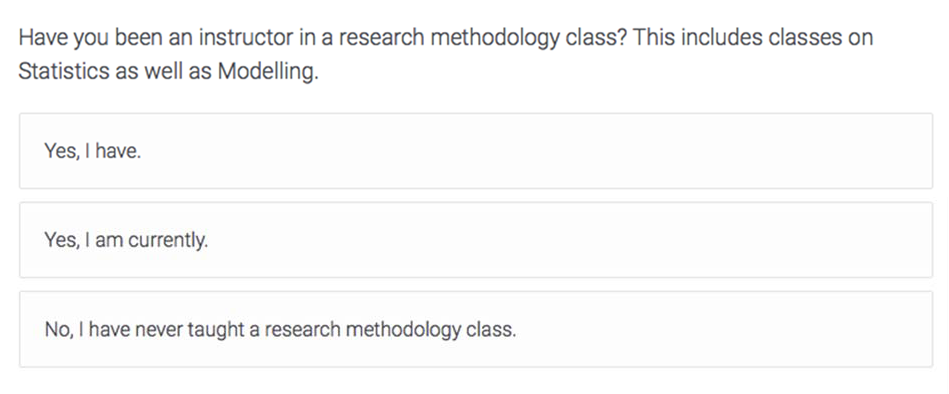

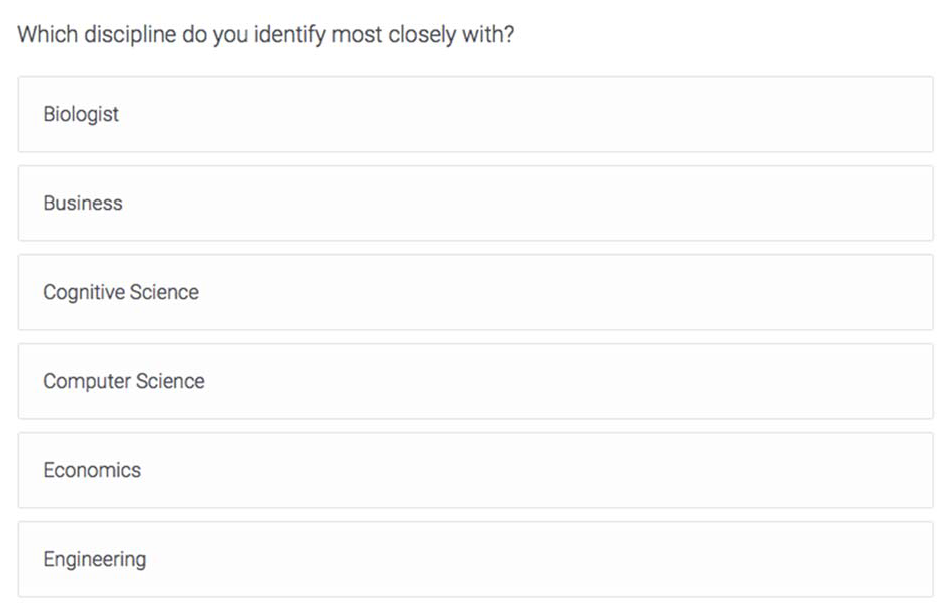

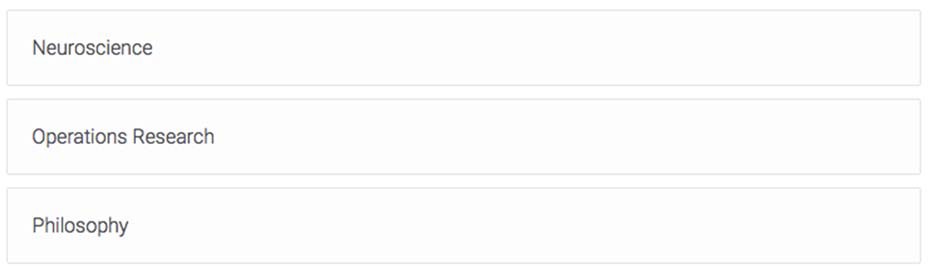

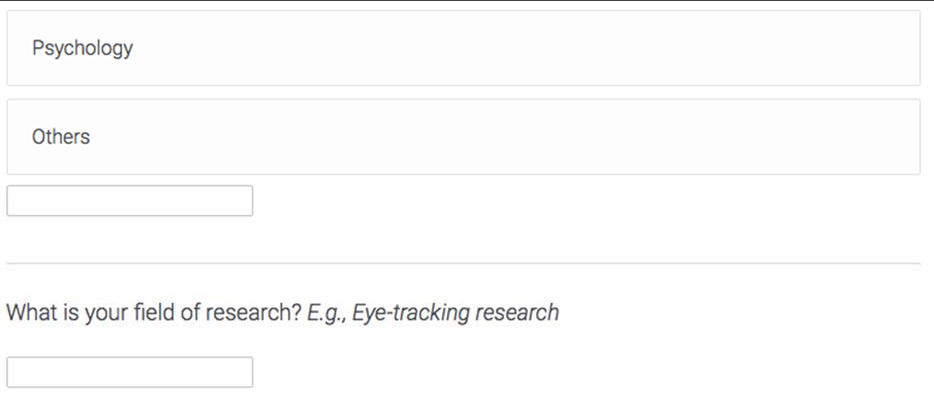

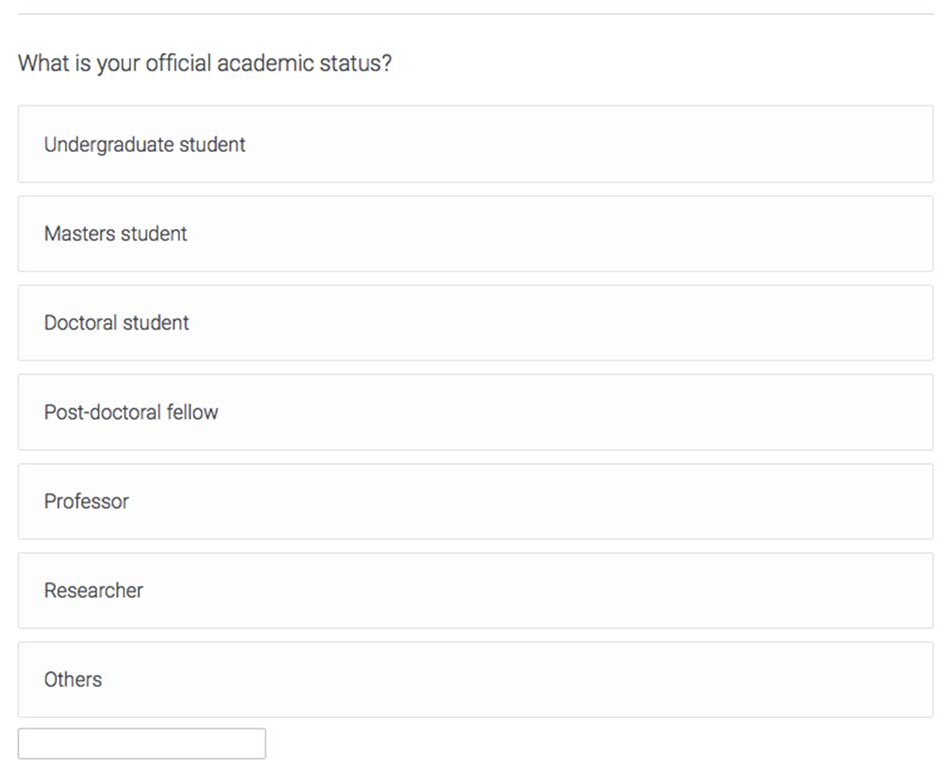

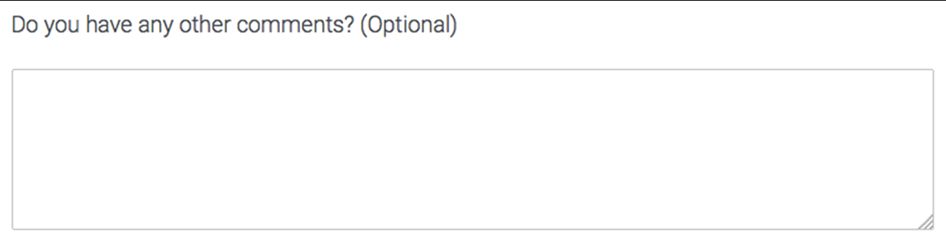


End of survey.

# Supplement D Application to a Mathematical Cognitive Model

The following supplement presents an exemplary application of the process model framework to a mathematical formal cognitive model of categorization. We apply the framework to the extended generalized context model (EGCM) by Lamberts (1998). To this end, we describe parts of the EGCM briefly. In the following, greek symbols denote free model parameters ($\lambda, \beta$) and roman symbols denote fixed parameters, or other model variables. The EGCM predicts categorization—the assignment of objects to categories based on object features *x_p_*—as a function of the perception of the similarity between a to-be-categorized exemplar and the previously-encountered exemplars. The perceived similarity $s_{ij}\left( t \right)$between an exemplar *i* and an exemplar *j*stored in memory encountered at time *t* is modelled by (Eq. 1)

$$s_{ij}\left( t \right)=\exp\left[ -\lambda\left( \sum_{p=1}^{P} I_{p}\left( t \right)u_{p}\left| x_{ip}-x_{jp} \right|^{r} \right)^{\frac{q}{r}} \right]$$

where *p* indexes the features of the exemplars, $I_{p}(t)$ is a binary indicator denoting if feature *p* was included in the computation at time *t*; $u_{p}$ is the utility of the feature dimension *p* in the classification*,* and $x_{ip}$ is the feature value of the *p*th feature of stimulus *i* (and correspondingly of stimulus *j* for $x_{jp})$. The parameter $\lambda$ is a free model parameter, representing discriminability in psychological space, with higher values leading to better discriminability. The exponent *r* determines the type of distance metric (*r=1* is Euclidean, *r = 2* is Manhattan distance), and *q* governs the transformation of distance into perceived similarity (exponential or Gaussian). Both *r* and *q* are often fixed to 1 or 2.

Regarding the inclusion of a feature dimension into the calculation, $I_{p}$, the model specifies the probability that at a certain point in time a feature of an exemplar is included in the categorization (given it has not yet been included) which depends on the perceptual salience of the stimuli and on the time: It is *I_p_(t)* = 1 – exp(–*q_p_t*), where *q_p_* is a inclusion rate for feature dimension *p*. This inclusion rate is assumed constant for each feature dimension. The inclusion probability is the cumulative probability from t=0 until t=t to include a feature dimension. Without time limits all feature dimensions can be included.

Given the computed similarity (based on including certain features at time t) the model predicts the category response, category *k*, given the feature combination $\boldsymbol{x}_{i}$of stimulus *i* as (Eq. 2)

$$\Pr\left( C_{k}| \boldsymbol{x}_{i} \right)=(1-g) \frac{\beta_{J} \sum_{j\in C_{j}} s_{ij}}{\sum_{k=1}^{m} {(\beta_{k} \sum_{k\in C_{k}} s_{ik}})}+g/m$$

where the parameter$\beta_{J}$ represents a bias towards selecting category *J* and $\beta$is a free model parameter; *k* indexes categories, the value $s_{ij}$ is the similarity between exemplars *i* and *j* from Eq. 1*,* and. The classification probability is the sum of the bias-weighted similarities of stimulus *s_i_* to the members of category *J*, which is standardized by the similarity of stimulus *s_i_* to all exemplars from all categories *k*. *g* is a guess rate and *m* is the number of categories.

To apply the process model framework to the EGCM, we will examine if the model has a scope with nested levels, contains one or more intermediate stages, its testability, separability, and compatibility.

The **conceptual scope** specifies *which model variables represent which properties of the cognitive system and sets the level of abstraction*. *Process models have nested levels of abstraction.* The similarity computation in the model is being related to the processing time of the cognitive system (p. 696), which relates model variables to properties of the cognitive system. Further, the EGCM hypothesizes two processing steps: “According to the EGCM, categorization of an object involves two consecutive steps. In the first step, perceptual processing of the stimulus takes place and the perceived similarity of the stimulus to stored exemplars is computed” (p. 696). This means that similarity computation is temporally nested between perception and choice. The model also has a temporal resolution, because the feature inclusion depends on *t.*

The **intermediate stage** is present if the model specifies *“variables at the nested intermediate level that directly or indirectly depend on the input variables and are not equal to the input variables (…) [and] produce the higher-level output variables”.* The EGCM theorizes that categorization involves the transformation of current and previously-seen feature combinations (input, ***x***_i_, ***x***_j_) into similarities *s_ij_* through a distance metric (Eq. 1). Therefore, the values of the model variable similarity are a function of the input. Further the computed similarity influences the categorization prediction (Eq. 2), this means the intermediate stage also influences the output.

**Testability** requires that models make *“testable predictions not only at the level of the output but also at the lower level of the intermediate stages.”* The EGCM’s process stage involves a similarity computation and memory retrieval (Eq 1). Two kinds of process predictions are made jointly with the classification prediction: one is about similarity and the other one about memory processes. The memory-retrieval prediction is an assumption of the model, which is not sufficiently precise to be operationalized (without making changes to the model). However, the prediction about the values of the similarity is precisely given by the intermediate stage variable *s_ij_* (Eq 1) which represents the psychological similarity, and is sufficiently precise to be operationalized and measured by other researchers. The model further predicts the category response. The testability of the EGCM’s processes is therefore also given.

Note that the free model parameters (discriminability $\lambda$, and category bias $\beta$) have a psychological interpretation and can in principle be correlated with psychological measures. However, because the parameters are free before these parameters are estimated, they do not yield precise predictions given the input to the model. If the parameter were fixed, these parameters would yield precise process predictions as well.

**Separability** involves that “*output predictions should not fully or partially produce the intermediate stage predictions (…) the intermediate stage variables do not decrease in their dependency on the input given the values of the output variables.”* In the case of the EGCM the model input (*x_ip_* and *x_jp_*, Eq. 1) produces both, the predicted psychological similarity *s_ij_* and the choice prediction (*Pr(C_k_* | *s_i_)* in Eq. 2), but the similarity values is not dependent on the output, after the free parameters have been estimated or have been fixed. Independent empirical support for the similarity predictions can, for instance, be obtained by measuring similarity beliefs. Support for the choice prediction may come from measuring category choices. As required by the separability criterion, similarity judgment data may support the similarity predictions of the model, whereas the categorization data might fail to support the predicted probabilities, and vice versa. Therefore, the model makes predictions that can be supported and refuted separately.

**Compatibility** entails *that the information transformations proposed in the intermediate stage of a process model are connected to the current understanding of cognitive capacities. (…) This can be a theoretical argument, an empirical argument, or a reference to data.* In his paper, Lamberts relates the similarity mechanisms that the model assumes to another psychological model, the generalized context model, by stating “This definition of similarity is closely related to the similarity definition in the GCM. (p. 696), which is a model with wide support in different domains.
